# Integrating Parental Reflective Functioning into Child and Adolescent Mental Health Services as a Pathway to Achieving SDG Targets: A qualitative study from South Africa

**DOI:** 10.1192/j.eurpsy.2026.11822

**Published:** 2026-07-31

**Authors:** Z. Noordien, T.-A. Adonis, M. Florence

**Affiliations:** Psychology, University of the Western Cape, Cape Town, South Africa

## Abstract

**Introduction:**

The global child and adolescent mental health crisis impedes achieving Sustainable Development Goal 3 (Good Health and Well-being) (Erskine et al., 2017). Parental Reflective Functioning (PRF), the capacity to mentalize a child’s internal world, is a key protective factor (Slade, 2005). However, its application in low-resource settings remains critically underexplored (Sharp et al., 2014). This study investigated service providers’ understanding of PRF in the Western Cape, South Africa.

**Objectives:**

This study aimed to explore how child and adolescent mental health service providers understand and apply the concept of PRF. The research focused on identifying their conceptualization of PRF, its practical application, and the barriers and facilitators to its implementation in clinical practice.

**Methods:**

A qualitative approach was employed, involving iterative workshops and semi-structured individual interviews with a multidisciplinary group of service providers who deliver support to children aged 10-19 who present with mental health challenges. The data were subsequently analysed using Braun and Clarke’s (Braun & Clarke, 2019) reflexive thematic approach to identify key themes related to PRF-informed practice.

**Results:**

Preliminary findings revealed a shift in provider attitudes towards recognizing the necessity of parental involvement. Key themes included: (1) Service utilization patterns often reveal a tendency for parents to externalize child and adolescent mental health challenges, positioning service providers as primary interveners rather than engaging as collaborative partners in the therapeutic process; (2) recognition of gaps in service provider training in relation to PRF and parent involvement (3) developing preliminary guidelines for “Optimal PRF” tailored to service providers.

**Conclusions:**

Integrating Parental Reflective Functioning is a central tenet of effective mental health promotion, not merely an adjunct to counselling. Strengthening PRF provides a scalable model for building resilient family systems, thereby directly contributing to the sustainable mental well-being goals outlined in SDG 3.4.

**Disclosure of Interest:**

None Declared

